# Long-term liver lesion tracking in contrast-enhanced ultrasound videos via a siamese network with temporal motion attention

**DOI:** 10.3389/fphys.2023.1180713

**Published:** 2023-06-26

**Authors:** Haozhe Tian, Wenjia Cai, Wenzhen Ding, Ping Liang, Jie Yu, Qinghua Huang

**Affiliations:** ^1^ School of Computer Science, Northwestern Polytechnical University, Xi’an, China; ^2^ Department of Interventional Ultrasound, Chinese PLA General Hospital Fifth Medical Center, Beijing, China; ^3^ School of Artificial Intelligence, Optics and Electronics (iOPEN), Northwestern Polytechnical University, Xi’an, China

**Keywords:** contrast-enhanced ultrasound, tracking, optical flow, kalman filter, motion, attention, template update, SIAMESE network

## Abstract

**Propose:** Contrast-enhanced ultrasound has shown great promises for diagnosis and monitoring in a wide range of clinical conditions. Meanwhile, to obtain accurate and effective location of lesion in contrast-enhanced ultrasound videos is the basis for subsequent diagnosis and qualitative treatment, which is a challenging task nowadays.

**Methods:** We propose to upgrade a siamese architecture-based neural network for robust and accurate landmark tracking in contrast-enhanced ultrasound videos. Due to few researches on it, the general inherent assumptions of the constant position model and the missing motion model remain unaddressed limitations. In our proposed model, we overcome these limitations by introducing two modules into the original architecture. We use a temporal motion attention based on Lucas Kanade optic flow and Karman filter to model the regular movement and better instruct location prediction. Moreover, we design a pipeline of template update to ensure timely adaptation to feature changes.

**Results:** Eventually, the whole framework was performed on our collected datasets. It has achieved the average mean IoU values of 86.43% on 33 labeled videos with a total of 37,549 frames. In terms of tracking stability, our model has smaller TE of 19.2 pixels and RMSE of 27.6 with the FPS of 8.36 ± 3.23 compared to other classical tracking models.

**Conclusion:** We designed and implemented a pipeline for tracking focal areas in contrast-enhanced ultrasound videos, which takes the siamese network as the backbone and uses optical flow and Kalman filter algorithm to provide position prior information. It turns out that these two additional modules are helpful for the analysis of CEUS videos. We hope that our work can provide an idea for the analysis of CEUS videos.

## 1 Introduction

CEUS requires the use of ultrasound contrast agents (UCA) to perfuse the lesion area. UCA in the vessels interacts with sound waves, producing a nonlinear harmonic response signal (Xu, 2009). With the assistance of contrast-specific imaging technology, CEUS can obtain the vascular distribution in the tissue and more microcirculation blood flow information than traditional ultrasound (Leen, 2001), which is particularly suitable for liver imaging due to the liver’s dual blood supply system (Brannigan et al., 2004). The CEUS process is typically divided into the arterial phase (AP), portal venous phase (PVP), and delay phase (DP) based on UCA perfusion time. Various categories of FLLs exhibit distinct enhancement and washout patterns in different phases (Liu et al., 2007). Therefore, accurate diagnosis of FLLs can be achieved by observing the enhanced state of the lesion tissue throughout the entire CEUS process. Nevertheless, focused observation for a long time increases the workload of physicians.

Currently, a wide variety of computer-aided diagnosis (CAD) systems have been created to help physicians categorize, segment, and diagnose medical images (Huang et al., 2021; Huang et al., 2022; Luo et al., 2022), particularly in ultrasound images (Huang et al., 2020a; Huang and Ye, 2021; Xu et al., 2022b; Li et al., 2022). Some research focuses on migrating research from the traditional imaging field to medical imaging (Xu et al., 2022a; Yan et al., 2022; Huang et al., 2023). Although CEUS has the benefit of producing no radiation or invasive problems (Beckmann and Simanowski, 2020), the quality of CEUS images might not always meet the requirements to train models for CAD systems. This is due to the presence of shadows and similar structures outside the target lesion area, causing significant noise interference. Furthermore, it is impractical to supply large numbers of video with manual annotations for model training due to the large number of frames during long-term scanning and the significant changes in texture and morphology. Consequently, owing to the limited quantity of CEUS images and the difficulty of accurate labeling, it is essential to track and extract the region of interest (ROI) of lesions in CEUS videos. Unfortunately, few researchers have recognized this issue. To address this problem, the most convenient approach is to refer to the studies on object tracking in traditional natural images.

In broad terms, object tracking is to determine the whereabouts of a designated object in subsequent frames with the given initial placement in the first frame. Correlation filter algorithms and siamese networks have emerged as the primary approaches to address this challenge. Correlation-based trackers accomplish this by resolving the ridge regression in the Fourier domain, which offers favorable adaptivity and efficiency, such as MOSSE (Bolme et al., 2010), CSK (Henriques et al., 2012), KCF (Henriques et al., 2014) and DSST (Danelljan et al., 2016). While correlation filter methods perform well for real-time tracking, they face many challenges such as scale variation, occlusion and boundary effects. Since 2016, siamese networks, like SiamFC (Bertinetto et al., 2016), SiamRPN (Li et al., 2018), have gained considerable traction by treating the tracking objective as a template matching operation. In general, current prevailing tracking methods can be summarized as a three-parts architectures, containing 1) a backbone to extract generic features, 2) an integration module to fuse the target and search region information, 3) heads to produce the target states. Siamese network is considered as the most popular pipeline for tracking. However, due to different features between medical images and natural images, general algorithms and models on the latter cannot be transformed well to deal with the former.

Meanwhile, many CAD analysis studies about CEUS keep appearing, but relatively few investigations have centred on object tracking in CEUS videos. Sirel et al. (Sirbu et al., 2022) evaluated a series of conventional trackers based on Boosting Algorithms and Analysis of optical flow, concluding that the KCF algorithm is suitable for CEUS imagery owing to its capacity to handle significant noise and low contrast. Wang et al. (Wang et al., 2020) developed a semi-automatic software that employs point-based registration techniques to track ROIs in CEUS cine-loops. While the software is user-friendly and efficient, its tracking efficacy is limited due to its reliance on key-point detection algorithms from MATLAB and its maximum frame limit of 400, which impairs its ability to process collected CEUS videos in real medical scenarios.

In real CAD scenarios, object tracking in CEUS videos is usually to better serve other research on medical diagnosis, such as benign and malignant classification and lesion segmentation. In many cases, obtaining the precise bounding box of a lesion is essential to discern its features. However, manual delineation in each frame can be arduous and time-consuming for physicians. Therefore, some researchers attempted to analyze only the optimal reference frames or focus on a specific position in the video within a short interval to extract image features (Seitz et al., 2010; Friedrich-Rust et al., 2013; Huang et al., 2020b). Nevertheless, these simplifications are inadequate to fully harness the underlying information present in CEUS sequences.

**FIGURE 1:**
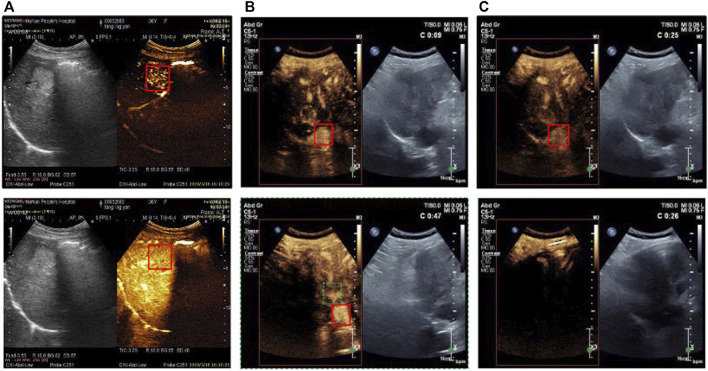
Examples of CEUS video frames (the target lesion is surrounding with the red solid line box) **(A)** Brightness of tumor region is increasing with the contrast agent coming from upper to lower **(B)** Tissue in the red dotted line box is similar to the real tumor in two frames **(C)** From 25 s to 26 s large area of shadow occurs in the video frames (from upper to lower).

With the rapid advancements in deep learning research, significant progress has been made in target object tracking in medical images such as B-mode ultrasound images, CT, MRI, and so on. However, tracking the target object, such as a liver lesion, in CEUS videos is different from traditional medical images because the features of the target object will undergo significant changes during the increase and dissipation of the UCA. This mainly manifests in changes in brightness and contrast with the background, as shown in Figure 1A. In most frames, the target object’s features are extremely inconspicuous, and some other tissues may even have features similar to those of the target lesion in previous frames, leading to misrecognition, as shown in Figure 1B. Furthermore, the lesion may experience short-term irregular displacement within a frame sequence due to the patient’s breathing or possible body movement. This can result in a large area of instantaneous shadow appearing in some frames, affecting the accuracy of traditional tracking algorithms, as illustrated in Figure 1C. These characteristics significantly increase the difficulty of long-term tracking and may even result in the misrecognition of the location area with large offsets.

In this work, we proposed a model based on the siamese network to assist track FLLs in long-term CEUS videos better. The main contributions of this work can be summarized as follows.

• We processed CEUS tracking tasks based on the idea of feature matching, used the classic siamese network as the backbone of the model, and mined the connotation of score map to provide the necessary basis for improving the module.
• Based on the motion trend information provided by optical flow, the Kalman filter method was used to model the focal motion system. We designed temporal motion attention to provide motion prior information to guide position prediction and reduce the influence of abnormal conditions in the long time tracking process.
• In view of the obvious changes in the features of lesions and surrounding tissues during CEUS, we designed a template updating mechanism for siamese network and updated strategies for possible abnormal situations, so as to better guarantee the effectiveness of template matching.

## 2 Methods

**FIGURE 2:**
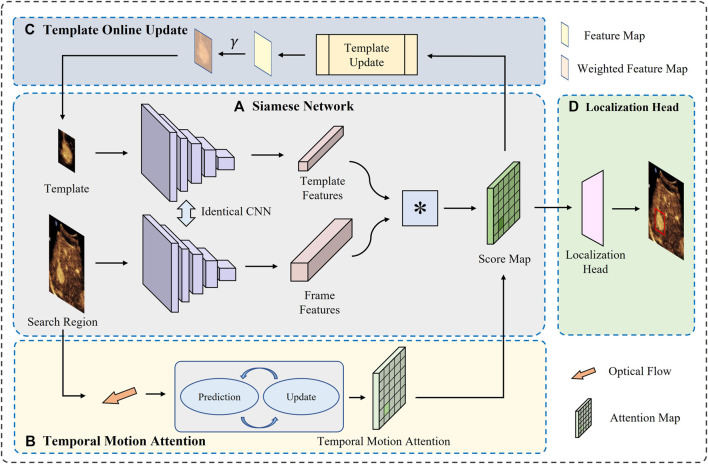
Overview of the proposed model. There are mainly four modules **(A)** The siamese network extract the features from both the template and search region in current frame, and then calculates the correlation between them **(B)** The temporal motion attention provides a prior knowledge about possible location based on optical flow **(C)** The template is updated online to satisfy the comparison **(D)** The eventual predicted location is generated by a head based on the integrated score map.

In this section, we describe the proposed framework in more detail. We first introduce the pipeline of the whole framework, and then present the three main modules. The whole model is shown in Figure 2.

The whole framework is based on siamese-branch structure. First, we train a siamese network on our collected CEUS images which have been validated by experienced doctors to extract features of the template and the search region in each frame. Then, for each frame, the optic flow between current frame and the last frame is calculated and modeled along with times to estimate the possible location of the target FLL. With the prior knowledge, a region can be cropped in current frame as the search region to put into the trained siamese network. With the template, a response map can be obtained for each frame, on which the maximum response region is considered as the location of the target FLL. During the process of the whole video, the template is updated online according to the correlation score between the template and the best-matching region in each frame. Finally, with the prior knowledge of position provided by motion estimation module and the constantly updated templates, the matching task is accomplished well.

### 2.1 Siamese model

As previously stated, the overarching concept of object tracking can be interpreted as a task of template matching between sequential frames. As for the location prediction, the model tackles it as a feature cross-correlation between the reference template and the candidate search regions. Moreover, to calculate the degree of likeness between the template and the candidate region in an iteration, a cost function is required, such as mean absolute difference (MAD), mean squared error (MSE), or cross correlation. This idea is exemplified by the remarkable achievements of siamese networks.

To elaborate further, siamese networks comprise two branches. The template branch is responsible for extracting the features of the tracked object from labeled images, which can then guide the search task in consecutive frames. The search branch, on the other hand, is tasked with extracting the features of the target search area within the current input image. The search area is typically a larger region of the potential location of the target. With the embedded features from each branch, a similarity score is computed using a correlation function, resulting in a score map. Finally, the position of the target is predicted by locating the best matching position in the score map.

As a pioneer of siamese network, the SiamFC network is primarily composed of two main branches, whereby an identical CNN is applied to both branches. The CNN is responsible for extracting representative embeddings in a common feature space for each branch, which is trained offline and evaluated online. The template branch takes the target region *z* in the initial frame as input, while the search branch takes a more extensive search area *x* in the current frame as input. A cross-correlation is then conducted between the two branches to quantify the degree of similarity.

$$s\left(z,x\right)=\phi \left(z\right)*\phi \left(x\right),$$ (1)

**FIGURE 3:**
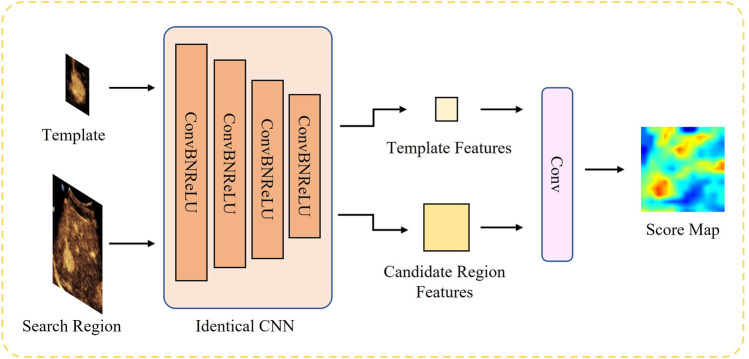
Structure of SiamFC backbone network. The features from the two branches are eventually fed into a correlation based on a convolution layer to generate the score map.

Where *ϕ*(⋅) is the identical CNN. Since the search region is larger than the template *z*, the output of this network is a score map corresponding to the number of candidate regions within the search region. The backbone of SiamFC is shown in Figure 3.

As mentioned earlier, training of SiamFC in was done in an initial off-line phase using the dataset of annotated videos. We selected a 5-layer full convolutional network for feature extraction, with channel numbers of 16, 32, 64, 128 and 64 respectively, and conducted training on the data set we collected. For details, please refer to section 3.1 and 3.3.

### 2.2 Temporal motion attention

As previously mentioned, SiamFC does not make any assumptions about object motion between consecutive frames. Consequently, the candidate frame (i.e., the current frame) is cropped using the previous target position as the center. It is evident that this assumption of a stationary object is not accurate, particularly for the US images, where there may be substantial motion between consecutive frames. Consequently, before the cropping phase, it is necessary to adjust the location of the target object. Therefore, we consider and analyze the motion state of the target to design the temporal motion attention, which provides *a priori* guidance for target positioning.

**FIGURE 4:**
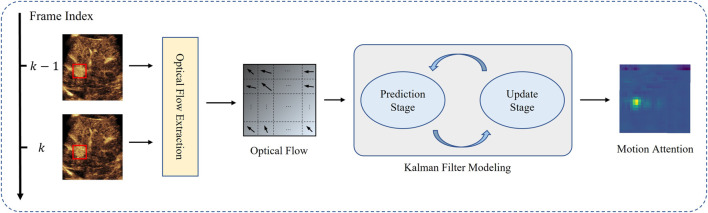
Temporal motion attention generated by optical flow and Kalman filter modeling.

Observing the CEUS videos obtained from actual scans, it can be found that lesion tissues typically undergo only positional translation. Therefore, we focus primarily on two aspects - the target’s motion speed calculation and state transition. Considering that CEUS images contain more light and shadow information compared to traditional US images, we use optical flow to compute the target’s movement direction, Then, we model the entire system by Kalman filter, and eventually predict the displacement with the temporal information. The whole pipeline is shown in Figure 4.

Let **u**(*t*) be the coordinates of the observable points in the image at time *t*, and $I\left(u\left(t\right),t\right)$ be the intensity of the points **u**(*t*). Since the tracking target moves in a very small range in a short time, the impact brought by the deformation and movement of the tissue is very small, and it can be considered that the observable points in the image are only displaced, while the intensity remains constant. After a very short time *δt*, the measured intensity of the corresponding pixels stays equal, that is, $I\left(u\left(t+\delta t\right),t+\delta t\right)=I\left(u\left(t\right),t\right)$ . Linearizing the log-intensity function *I* of time using the first-order Taylor approximation yields

$$I\left(u\left(t+\delta t\right),t+\delta t\right)\approx I\left.\left(u\left(t\right),t\right)\right)+\frac{\partial I}{\partial u}\frac{\partial u}{\partial t}\delta t+\frac{\partial I}{\partial t}\delta t.$$ (2)

Hence, we can obtain the constraint for the intensity change

$$\frac{\partial I}{\partial t}\left(u\left(t\right),t\right)+\nabla I\left(u\left(t\right),t\right)\dot{u}\left(t\right)=0,$$ (3)

Which associates the change in intensity over time with the spatial change in intensity over the displacement of points. The term $\nabla I\left(u\left(t\right),t\right)$ is regarded as the optical flow at time *t*.

Furthermore, the change in intensity over a short time interval Δ*t* can be approximated as follows.

$$\Delta I\left(u,t\right)=I\left(u,t+\Delta t\right)-I\left(u,t\right)\approx \frac{\partial I\left(u,t\right)}{\partial t}\Delta t.$$ (4)

According to Eq. 3, 4 the intensity change can be rewritten as

$$\Delta I\left(u,t\right)\approx -\nabla I\left(u,t\right)\dot{u}\Delta t,$$ (5)

Which explains the intensity change Δ*I* generated by the flow displacement of points with $\dot{u}$ along the intensity gradient ∇*I* (**u**, *t*) in the time interval Δ*t*.

Therefore, in turn, by calculating the optical flow information and intensity changes of the image, the displacement of points can be obtained. In order to calculate the optical flow, Lucas–Kanade (LK) algorithm is applied.

The LK optical flow method is an efficient algorithm to calculate the optical flow between two consecutive frames. In a real continuous tracking scene, the distance of observation points moving between adjacent frames is small. According to the assumption of spatial coherence of optical flow, the neighboring observation points in the image have similar motion behavior in the local range. Therefore, the velocity value of the central pixel can be calculated based on the surrounding pixels. A set of equations can be established based on Eq. 5 to describe the points *p*_*i*_ (*i* = 1, … , *n*) belonging to a observation window in the 2D ultrasound image

$$\underset{U}{\underbrace{\left[\begin{array}{cc} I_{x}\left(p_{1}\right) & I_{y}\left(p_{1}\right)\\ I_{x}\left(p_{2}\right) & I_{y}\left(p_{2}\right)\\ \vdots & \vdots \\ I_{x}\left(p_{n}\right) & I_{y}\left(p_{n}\right) \end{array}\right]}}\left[\begin{array}{c} u_{x}\\ u_{y} \end{array}\right]=\underset{V}{\underbrace{\left[\begin{array}{c} -I_{t}\left(p_{1}\right)\\ -I_{t}\left(p_{2}\right)\\ \vdots \\ -I_{t}\left(p_{n}\right) \end{array}\right]}},$$ (6)

which explains that the optical flow of this observation window can be obtained by observing and tracking some selected neighboring points in the window. Applying the least squares method, the above equation can be solved

$$\left[\begin{array}{c} u_{x}\\ u_{y} \end{array}\right]={\left(U^{⊤}U\right)}^{-1}\left(U^{⊤}V\right).$$ (7)

The LK algorithm is also referred as the local flow estimation, which only focuses the local movement and can avoid the global error propagation. In real practice, each frame is divided in small patches to satisfy the assumptions of the same brightness and smoothness. By solving a system of linear equations based on the constant intensity model, the velocity vector of each pixel in the image grid can be calculated.

In theory, the trajectory of a rigid body’s motion in a short period of time can be viewed as a smooth curve, often exhibiting regular back-and-forth movement due to the patient’s breathing. However, in actual clinical scenarios, the motion trajectory of the target may experience some jitter due to the physician’s unstable scanning technique, especially when encountering shadows, similar backgrounds, or other interferences, which may cause tracking failures. To address this issue, we model the entire measurement system using a Kalman filter method, which incorporates the motion direction information provided by optical flow to correct and predict the target displacement.

Let us denote $x\left(t\right)={\left[u\left(t\right),v\left(t\right)\right]}^{⊤}$ as the state of the target tracking area, where $u\left(t\right)={\left[a_{x}\left(t\right),a_{y}\left(t\right)\right]}^{⊤}$ and $v\left(t\right)={\left[v_{x}\left(t\right),v_{y}\left(t\right)\right]}^{⊤}$ are the position and velocity of particle at time *t*, respectively. Considering the actual displacement as a linear system with Gaussian noise, the state model of the target can be expressed as

$$x\left(t\right)=A_{t-1}x\left(t-1\right)+\omega_{t-1},$$ (8)

Where **A**_*t*−1_ is the state transition from **x**(*t* − 1) to **x**(*t*), and *ω*_*t*−1_ ∈ *N* (0, **Q**) is the process noise with the covariance **Q**.

For every detection measurement of **x**(*t*), the obtained state is

$$z\left(t\right)=H_{t}x\left(t\right)+\sigma_{t},$$ (9)

Where **H**_*t*_ is the measurement transition from true state to the measured state, and *σ*_*t*_ ∈ *N* (0, **R**) is the measurement noise with the covariance **R**.

After the initial state estimation, let the covariance $P_{t}^{-}$ denote as a noise distribution to measure the reliability of the Kalman filter final state estimation. The parameter should be updated by covariance at time *t* − 1

$$P_{t}^{-}=A_{t-1}{\hat{P}}_{t-1}A_{t-1}^{⊤}+Q$$ (10)

Next, the Kalman gain *K*_*t*_ at time *t* is calculated based on the covariance of the prediction results and the uncertainty *R* of the observation process as follows:

$$K_{t}=P_{t}^{-}H_{t}^{⊤}{\left(H_{t}P_{t}^{-}H_{t}^{⊤}+R\right)}^{-1}$$ (11)

After obtaining the Kalman gain **K**_*t*_ at time *t*, the estimated state is updated as

$$\hat{x}\left(t\right)=\hat{x}\left(t-1\right)+K_{t}\left(z\left(t\right)-H_{t}{\hat{x}}^{-}\left(t\right)\right)$$ (12)

Finally, the noise distribution of the estimation is updated

$${\hat{P}}_{t}=\left(I-K_{t}H_{t}\right)P_{t}^{-}$$ (13)

By using Kalman filter to model the measurement process of the system, the predicted system state, namely, the displacement and motion state of the focus, can be obtained. Based on this, we use Gaussian probability model to model around the predicted location

$${\text{Att}}_{M}=\exp-\frac{d_{i}^{2}}{2\sigma^{2}}$$ (14)

Where $d_{i}={\left\|l_{i}-l_{c}\right\|}_{2}$ represents the moving distance between the location of point *p*_*i*_ and the location of the predicted location *p*_*c*_ in the current frame. *σ* is the standard deviation of the moving distance of the objects in 10 frames before the current frame. Take the facts into account, we limit *σ* between 3 and 9, for which too small variance will cause the judging strategy of the object movement to be very conservative, while too large variance will make the judging strategy invalid. In addition, we set the probability threshold to 1e-4, and probabilities lower than the threshold are deemed invalid.

The application of this mechanism can adaptively limit and correct the object position of current frame according to object’s movement state in current time, solving the problem of misrecognition and permanent object loss.

### 2.3 Online adaptive template update

SiamFC is a tracking model based on the idea of similar matching, which can predict the movement of the target by looking for the position that is most similar to the template feature in the candidate region of searching branch. Therefore, the tracking effect of this network depends heavily on the validity of the template. If the template features do not reflect the tracking target effectively, the matching will be invalid, resulting in the loss of the target. In the process of CEUS, the filling and fading of contrast agent will make tissues and organs show dynamic process of light and dark changes with the assistance of imaging technology. Therefore, if the image marked in the first frame is always used as a template, it will not be able to represent the target that changes in light and shade later. This challenges the tracking model. Therefore, to solve this problem, we design a template adaptive update mechanism to constantly update the image of the template branch.

The conventional template linear update approach employs fixed update weights to update the weights. Despite its limitations, this strategy has been the norm for online updating due to its acceptable results (Zhang et al., 2019). However, evidently, this approach continuously weakens the influence of the true target features in the first frame, potentially leading to severe template pollution issues in subsequent frames and causing the tracking performance to deteriorate over time. This issue is particularly pronounced in long-duration video object tracking scenarios. In accordance with the mechanism proposed above, we have made a simple improvement to the conventional template online updating approach.

Considering that the purpose of template updating is to better guide the matching, we set a coefficient *γ* related to the similarity between the two feature maps as the weight of the linear superposition of features

$$\hat{T}=\left(1-\gamma \right){\hat{T}}^{-}+\gamma Z_{i-1},$$ (15)

where ${\hat{T}}^{-}$ is the previous template, and $Z_{i}$ represents the template generated from the *i*th frame. *γ* is a evaluation criteria for the similarity between the template and the current frame. It depends on the intensity of the pixels in the images. As for the diagnosis using CEUS videos, a curve measuring intensity along time, called TIC, is often used as a crucial criteria. *γ* is the growth rate of image intensity in the two region, which is adaptive to individual videos according to the TIC.

Another important issue with template updates is determining whether the update operation needs to be triggered. In general, we chose to update the template every 5 frames, considering that the actual videos usually have an FPS of 15. At the same time, we have developed strategies to deal with abnormal situations that often occur during the long tracking process.

Due to the complexity of the actual collection, the quality of the collected video is mixed. Therefore, before the experimental test, we fully considered several common situations and carried out targeted detection processing, in order to expect that the proposed model can be better applied in the actual diagnosis scenario. Invalid frames produced for short periods of darkness can contaminate the original feature if template updates are also performed. Therefore, in view of this situation, we recorded the intensity of the moving area of the lesion in the image during the tracking process, like the TIC recording process. When the overall intensity was lower than the threshold *η*, abnormal conditions were indicated, and tracking and template updating were not carried out at this time. After the intensity rise image was recovered, the location of the lesion was repositioned according to the previously calculated motion information.

Through this simple improvement, we can continuously absorb key useful features, and reduce the pollution of ineffective features.

## 3 Experiments

### 3.1 Datasets

To examine the proposed model, we obtained 33 CEUS videos with a total of 37,549 frames from our partner medical institutions. All the videos were collected under the recommendations of the CEUS parameters from related manufacturers as a reference. Under the guidance of the standard protocol (Dietrich et al., 2020), CEUS examines were performed by a convex probe and a dual screen format with low-mechanical index, after a bolus injection of 2.0–2.4 mL of SonoVue (Bracco SpA, Milan, Italy) in the antecubital vein and a following flush by a 5-mL saline. From the time the bubble first appeared until 120 s after injection, the CEUS cine loops were constantly captured. Once the microbubbles had cleared entirely from the index lesion, the lesion was sporadically scanned and filmed in 5-s cine loops every 30 s for 5 min. All imaging data was stored in DICOM format. All the images are 800 × 600 and then cropped out the CEUS area according to the coordinate labels in DICOM raw file.

All videos were annotated by medical professionals. During labeling, the first frame was taken as the moment when the lesion site first appeared in the visual field. After that, the location of the lesion was marked every 10 frames, amd finally a total of 3,524 annotated frames were obtained. In addition, to meet the model training requirements, we used data from an additional 875 cases, each containing 7 CEUS images (3 frames in AP, 2 frames in PVP and 2 frames in DP) with annotated information. All data were annotated and checked by two or more physicians.

### 3.2 Evaluation metrics

To comprehensively evaluate the proposed network segmentation performance, we use three different evaluation metrics, namely, TE, RMSE, IoU and FPS. TE and RMSE measure location accuracy and tracking robustness from both horizontal and longitudinal perspectives. IoU evaluates accuracy from the perspective of actual tracking effect. FPS measures how efficiently a model is executed.

**TE.** Given the ground-truth annotations *p*_*j*_ and tracked outputs ${\hat{p}}_{j}$ , the tracking error for a given target *i* is calculated as

$${\text{TE}}_{j}^{\left(i\right)}\left(t\right)=\left\|p_{j}-{\hat{p}}_{j}\right\|,$$ (16)

where ‖ ⋅‖ represents the Euclidean distance between the estimated landmark position *p*_*i*_ and its ground-truth annotation ${\hat{p}}_{i}$ .

**RMSE.** In order to comprehensively evaluate the overall deviation, we calculate the average RMSE between the centroid of the predicted bounding box and that of the ground-truth. The RMSE is calculated both in the lateral and the axial directions as well. For the *i*th video, the RMSE along the direction *k* is calculated as

$${\text{RMSE}}_{k}^{\left(i\right)}=\sqrt{\frac{1}{M^{\left(i\right)}}\underset{M^{\left(i\right)}}{\sum }{\left({\hat{p}}_{k;j}-p_{k;j}\right)}^{2}},$$ (17)

Where *M* ^(*i*)^ is the total number of labelled frames in video *i*. ${\hat{p}}_{k;j}$ represents the predicted horizontal or vertical coordinates in the *j*th frame, and *p*_*k*;*j*_ is the groundtruth label.

**IoU.** While TE and RMSE are important for localization, larger values of IoU are desirable for accurately enclosing the carotid artery. IOU is defined as the intersection between the predicted and ground-truth bounding boxes. We calculate the mean IOU of each video and get the average mIoU as the metric for the accuracy of bounding box. The IoU for the *i*th video is formulated as

$${\text{IoU}}^{\left(i\right)}=\frac{1}{M^{\left(i\right)}}\underset{M^{\left(i\right)}}{\sum }\frac{{\hat{G}}_{j}\cap G_{j}}{{\hat{G}}_{j}\cup G_{j}},$$ (18)

Where ${\hat{G}}_{j}$ is the predicted bounding box in the *j*th frame, and *G*_*j*_ is the groundtruth.

**FPS.** Another important evaluation metric on whether the model can really be applied to real-world scenarios is the processing speed. So, we record the processing time and calculate average FPS, and then compare it with the original frame rate of each video. In this way we can test whether the model processing can deal with online tasks.

### 3.3 Implementation details

In terms of the siamese network, the model was implemented with pytorch and was trained and tested on a server using one single TITAN RTX GPU on Ubuntu 20.04.2 LTS platform. We use a SGD optimizer with 0.9 of momentum and 5e-4 of weight decay for training. Based on initial empirical tests, we employed a batch size of 8 video clips, a learning rate of 1e-7, and trained for 200 epochs with the Adam optimizer. We explored the collected videos and selected 0.7 as the coefficient of the template module to update the template continuously.

### 3.4 Comparative study

We compared our results with existing generic methods. Since there are few related works about lesion tracking in CEUS videos, we compared our proposed framework with some classical models commonly used in other medical images. CSK (Henriques et al., 2012), KCF (Henriques et al., 2014) and DSST (Danelljan et al., 2016) are the classical correlation filter algorithms. SiamFC (Bertinetto et al., 2016) and SiamRPN (Li et al., 2018) are the most popular siamese networks nowadays. All models were replicated in the comparison experiments section using the official code. To ensure fair comparisons among the models, no pretraining weights were employed for any of them.

As shown in Table 1, our model produced the mIoU of 86.43%, higher than the results of other methods. The smallest TE of 19.2 pixels and RMSE of 27.6 demonstrates that the trajectory predicted by our framework is smoother which exactly corresponds to the generally regular movement of the FLLs. Although the FPS of the model has decreased due to the introduction of additional modules, it can basically meet the practical auxiliary medical applications.

**TABLE 1:** Quantitative comparison of different generic methods and models. The methods contains the related work and other classical models.

Methods	mIoU (%)	TE	RMSE	FPS
Wang et al. (2020)	69.57	26.7	110.4	4.35 ± 1.41
CSK	55.89	45.3	136.2	32.67 ± 2.48
KCF	81.44	24.6	53.8	30.51 ± 8.43
DSST	80.36	22.1	49.5	17.51 ± 4.26
SiamFC	71.63	21.2	67.5	19.42 ± 3.81
SiamRPN	72.34	19.8	70.4	13.23 ± 2.17
Ours	86.43	19.2	27.6	8.36 ± 3.23

**FIGURE 5:**
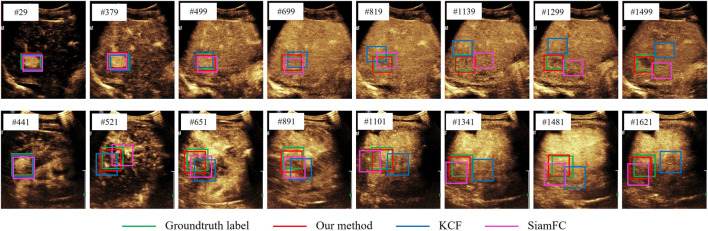
Visualization of segmentation results for different models.

Figure 5 shows the tracking results of our model and other models. We can see that at the beginning of the tracking stage, the background has not been filled with contrast agent, so the focus and background are clearly distinguished, and the tracking effect of most models is good. However, as the background gradually brightened, the feature difference between the lesion and the background became smaller and smaller, and the detection-based tracking method SiamFC could no longer accurately find the target. After the transient loss of the target, the KCF algorithm, which is strictly based on the previous frame, presents the prediction frame drift problem, which is due to the pollution of the target features caused by forced matching. Due to the use of temporal motor attention, our model models the movement trend of the focus, so that when target loss occurs, the original target can be found to a certain extent under the guidance of attention.

### 3.5 Ablation experiments

To illustrate that the core modules in the proposed network are effective, we conduct ablation experiments of the proposed framework on our dataset. As shown in Table 2, we drop the proposed two additional modules respectively in the proposed network and measure the tracking metric of the remaining model.

**TABLE 2:** The ablation experiments of the proposed model. These mainly includes the experiments of the temporal motion attention (TMA) module and the template update (TU) module.

Methods	mIoU (%)	TE	RMSE	FPS
SiamFC	71.63	21.2	67.5	19.42 ± 3.81
SiamFC + TMA	80.47	20.2	35.9	10.15 ± 2.31
SiamFC + TU	81.61	19.6	54.7	17.58 ± 2.71
Ours	86.43	19.2	27.6	8.36 ± 3.23

Table 2 demonstrates that the proposed model has the greatest impact on network performance due to the two designed modules. The TMA module captures the motion law of the lesion area and provides the location prediction probability, which provides another mode prior knowledge guidance for the selection of the search area. Thus, the whole motion pattern is smoother and more in line with the actual situation, so that the RMSE decreases. The TU module constantly updates the template to ensure that the template features are as close as possible to the features of the focal area in the current frame, thus maintaining the premise assumption of the tracking model to a certain extent, as a result the tracking error decreases.

**FIGURE 6:**
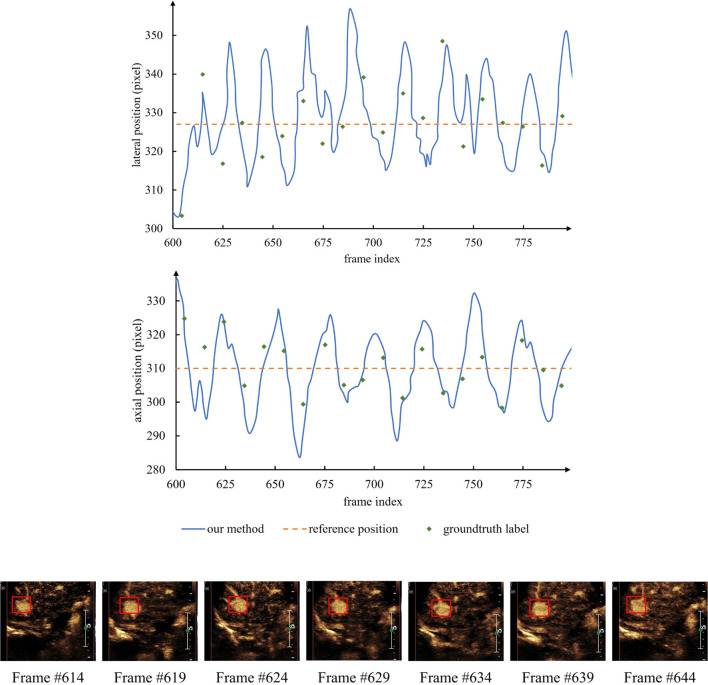
An example showing the reciprocating motion of the lesion. The actual numerical statistics can prove the hypothesis of the temporal motor attention model, and further verify the validity of the model.

To further demonstrate the effectiveness of temporal motor attention, we recorded the movement trajectories of focal centers. Figure 6 represents an example of tracking a landmark in a randomly chosen image sequence from the dataset. The two graphs represent the displacement of the landmark along with lateral and axial directions, respectively, for a set of consecutive frames. Landmark locations obtained by our framework, ground truth, and the no tracking methods are plotted. For better visualization of the regular movement, we also display a set of images with annotations for the landmark location obtained by the ground truth, our framework, and no tracking methods. The locations corresponding to the annotations in these images are also plotted in the two graphs.

## 4 Discussion

In this paper, two modules are designed on the basis of siamese network, so that the tracker can maintain good tracking effect in the complex scene of CEUS videos to some extent. Temporal motion attention uses optical flow information to predict the displacement by calculating the movement trend of the target, and uses Kalman simulation to measure the motion state of the target in the presence of noise, hoping to better fit the real scene. Attention provides priori information for the location of the lesion and guides the determination of the target location in the score map. In the process of CEUS treatment, the characteristics of the lesion and its surrounding tissues showed obvious characteristic changes with the filling and fading of contrast agent. Considering this feature of CEUS video, we designed a template adaptive update mechanism, which updates the template features gradually with the help of score map reflecting the matching situation. It turns out that template updating mechanism is very necessary for CEUS video analysis.

According to the experiment results, the proposed modules have provided a solution to solve the mentioned problems in Figure 1. The images of changing intensity are fed into the model and extracted the target area as the template continuously. The TU module ensures the validity of matching between template and the current frame. The TMA module provides a prior instruction to locate the target and narrow the search area. Therefore, the identification of similar targets and the target disappearance due to shadows existence can be solved. These modules enhance the method to select the candidate search region.

In our initial study, we tried the classical siamese architecture model in the field of target tracking, but eventually found that the siamese family of methods was not as effective as the KCF algorithm (as shown in Table 1). The KCF algorithm searches each frame based on the position of the target in the previous frame, while the basic siamese model is based on the initial template, which makes it difficult to accurately match the target position when the target brightness and color are constantly changing. This makes it difficult to match the target position accurately when the target brightness and color are changing. However, due to this feature of KCF algorithm, in the case of short-time darkness, the correct target features will be lost due to the forced matching, and finally the target will be lost. Therefore, this paper adds two modules to the SiamFC network in order to better integrate the advantages of the two methods.

As for the RPN-based siamese models, we think the bad performance mainly attributes to the following two reasons. First, the RPN module is a universal target detection structure based on the idea of classification. Thus, the generated candidate anchors themselves may be inaccurate, and as a result, the best candidate frames after selection are definitely not accurate enough. In addition, during the detection of each frame, the RPN module performs generally independently, which can handle rapid changes for traditional images. But for CEUS videos, where the shape of the FLL changes little and the position moves regularly, it cannot give full play to its advantages, and instead, due to the independence of each frame detection, the mutual reference information is lost. Consequently, the location of the all predicted bounding boxes moves not smoothly.

At present, our model mainly deals with the three main abnormal situations observed, adds the template updating mechanism for the feature changes to ensure the establishment of the tracking hypothesis, and introduces the motion information of the target to guide the disappearance of the target. In view of the lack of attention in the current research on this issue, the model we proposed is also a preliminary attempt at present, and there are still many problems that need to be improved in the future. For example, in the portal pulse stage, the lesion area is too similar to the background, which is difficult to effectively locate. Because the quality of the collected video cannot be guaranteed, it is recommended to evaluate the quality of the video in advance before the actual application. As we all know, applying a designed model to a real world scenario requires sufficient robustness of the model, and this part needs to be further improved.

## 5 Conclusion

In this article, we addressed two major limitations of the siamese architecture-based object tracker on CEUS videos. By introducing the template update module, we resolved the constant position model issue and improved the robustness of SiamFC against deforming landmarks. We mined the motion law of the focus, aiming at the difficulty of location in the multi-similar background, modeled the motion state of the focus using Kalman filter method based on the optical flow information, and finally introduced the time sequence motion attention to guide the location prediction. Our proposed model achieved an overall mean IoU of 86.43% that is comparable to other baseline methods. The whole framework also provided promising results against synthetically induced occlusions demonstrating the potential for accurate and robust landmark tracking. For our future work, we intend to improve the detection module of the siamese network. Using region proposals along with siamese architecture and combining it with the two modules introduced in this article could significantly improve tracking accuracy. In addition, we also intend to develop nonlinear motion models tailored to the needs of specific FLL motion.

## Data Availability

The original contributions presented in the study are included in the article/supplementary material, further inquiries can be directed to the corresponding authors.
